# Recent Progress of Sub‐Nanometric Materials in Photothermal Energy Conversion

**DOI:** 10.1002/advs.202104225

**Published:** 2021-11-27

**Authors:** Qichen Lu, Xun Wang

**Affiliations:** ^1^ Key Lab of Organic Optoelectronics and Molecular Engineering Department of Chemistry Tsinghua University Beijing 100084 China

**Keywords:** biomedicine, hydrothermal/solvothermal methods, light‐responsive composites, photothermal conversion, solar vapor generation, sub‐nanometric materials

## Abstract

Sub‐nanometric materials (SNMs) are an attractive scope in recent years due to their atomic‐level size and unique properties. Among various performances of SNMs, photothermal energy conversion is one of the most important ones because it can efficiently utilize the light energy. Herein, the SNMs with photothermal energy conversion behaviors and their applications are reviewed. First, a hydrothermal/solvothermal method for the synthesis of SNMs is systematically discussed, including the LaMer pathway and the cluster–nuclei coassembly pathway. Based on this synthetic strategy, many kinds of SNMs with different morphologies are successfully prepared, such as nanorings, nanowires, nanosheets, and nanobelts. These SNMs exhibit excellent photothermal performance under the laser or solar irradiation according to their different light absorption ranges. These enhanced absorption performances of SNMs are induced by the mechanism of plasmonic localized heating or nonradiative relaxation. Finally, the applications of the photothermal SNMs are illustrated. The SNMs with photothermal behaviors can be widely applied in the fields of solar vapor generation, biomedicine, and light‐responsive composites construction. It is hoped that this review can provide new viewpoints and profound understanding to the SNMs in photothermal energy conversion.

## Graphical abstract

The recent progress of sub‐nanometric materials in the field of photothermal energy conversion is reviewed, including the synthesis methods, photothermal performance under laser or solar irradiations, and potential applications, such as solar vapor generation, biomedicine, and light‐responsive composites construction. This review aims to provide reference for the further development in this field.

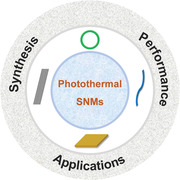

## 1 Introduction

Nanomaterials, which refer to materials with at least one dimension in the scale between 1 and 100 nm,^[^ ^1^ ^]^ have attracted extensive attentions due to their unique properties. In the last few decades, many kinds of nanomaterials have been fabricated successfully, including metals,^[^ ^2^ ^]^ semiconductors,^[^ ^3^ ^]^ carbon‐based materials,^[^ ^4^, ^5^ ^]^ MXenes,^[^ ^6^ ^]^ etc., and they are widely applied in the fields of catalytic reactions,^[^ ^7^, ^8^, ^9^ ^]^ energy storage,^[^ ^9^, ^10^, ^11^ ^]^ energy conversion,^[^ ^12^, ^13^ ^]^ biomedicine,^[^ ^14^, ^15^, ^16^, ^17^ ^]^ etc. Among all the factors which can influence the properties of nanomaterials, the size and shape of nanomaterials are crucial ones and have been studied by researchers in depth. For example, Ag nanomaterials with different sizes and shapes will show different plasmonic properties.^[^ ^18^ ^]^ However, there is still a transition region between traditional nanomaterials and molecules, in which the size of the materials is around or even less than 1 nm. According to the previous work, the above mentioned nanomaterials are suggested to be defined as sub‐nanometric materials (SNMs).^[^ ^19^ ^]^ Specifically, for the meaning of SNM, two aspects need to be required:^[^ ^19^, ^20^ ^]^ the first one is that the size of SNMs should be at a single‐unit cell level or molecular level, which is usually at nearly 1 nm, and the other one is that the sized‐related properties of SNMs are quite different from that of conventional nanomaterials.

Based on different morphologies, the SNM can be divided into three types:^[^ ^20^ ^]^ 0D SNM, 1D SNM, and 2D SNM. Clusters, such as polyoxometalates (POMs), can be regarded as 0D SNM. They usually have precise chemical compositions, and even the change of one atom will influence their properties.^[^ ^21^, ^22^ ^]^ 1D SNM contains the morphology of wires, belts, and tubes. As a typical example, sub‐nanometric wires (SNWs) show novel properties due to their single‐unit‐cell diameter. Specially, the size and dimension of SNWs are similar with those of polymers, and the SNWs have some polymer‐like features,^[^ ^19^, ^23^ ^]^ such as mechanical flexibility, self‐assembling, and gelation phenomena, which are quite different from conventional inorganic nanomaterials. Thus, the SNWs can also be called “polymer‐analogue.” As for 2D SNM, layered nanosheet structures are belong to this scope, such as graphene,^[^ ^4^, ^24^ ^]^ layered double hydroxides (LDHs),^[^ ^25^, ^26^ ^]^ transition metal dichalcogenides,^[^ ^27^, ^28^ ^]^ black phosphorus,^[^ ^29^, ^30^ ^]^ MXenes,^[^ ^6^, ^31^ ^]^ metal–organic frameworks,^[^ ^32^, ^33^ ^]^ and covalent organic frameworks.^[^ ^34^, ^35^ ^]^ Because of the atomic level size, the SNMs have large surface area, which facilitates the loading of other components, especially for 2D SNMs.^[^ ^36^ ^]^ Meanwhile, the SNMs have high ratio of surface atoms, therefore they exhibit high‐effective atom utilization in catalytic reactions^[^ ^37^ ^]^ and energy conversion processes.^[^ ^19^ ^]^

Photothermal energy conversion is an important method to utilize light energy. In this field, photothermal materials first absorb the light energy, and then convert it into heat energy for further use. According to the wavelength range of the light source, the photothermal process can be divided into two categories. i) Near‐infrared (NIR) laser serves as the light source. The wavelength range of NIR light is ≈780–2500 nm, which is between visible light and medium infrared light. Due to its advantages of weak tissue attenuation and small tissue damage, NIR light has been widely used in biomedicine, including photothermal therapy (PTT)^[^ ^15^, ^16^, ^17^, ^38^, ^39^, ^40^, ^41^, ^42^, ^43^, ^44^ ^]^ and photoacoustic (PA) imaging.^[^ ^45^ ^]^ For example, when injecting a photothermal material into the tumor site and using an NIR laser to irradiate the tumor area controllably, the tumor cells can be selectively ablated.^[^ ^28^ ^]^ ii) Solar light irradiation is applied as the energy source. Solar energy has advantages of wide distribution, large capacity, and cleanness, so it is a promising renewable energy source.^[^ ^46^ ^]^ Among various technologies for utilizing solar energy, solar‐to‐thermal conversion process is a direct and efficient way. For instance, in a solar vapor generation process, the energy efficiency can reach more than 90%.^[^ ^47^, ^48^, ^49^, ^50^ ^]^ In the whole photothermal conversion system, light absorbing materials are the most important part. They are required to show strong light absorption capacity, as well as rapid photo‐to‐thermal conversion behaviors. Therefore, the design of light absorbing materials is crucial for the application of photothermal energy conversion.

Here, we focus on the synthesis of SNMs and their applications on photothermal energy conversion field. First, the synthesis of SNMs based on hydrothermal/solvothermal methods is illustrated, including the traditional LaMer pathway and the cluster–nuclei coassembly pathway. Then, the photothermal conversion performance of SNMs and corresponding mechanism are demonstrated. Next, the applications of SNMs in photothermal energy conversion, including solar vapor generation, biomedicine, and light‐responsive composites construction, are briefly discussed. Finally, a summary and some challenges are proposed in this field. We hope that this review can facilitate researchers deeply understanding the synthesis and photothermal conversion behaviors of SNMs, and further designing novel SNMs with enhanced performance.

## 2 Synthesis of SNMs

Many strategies have been developed to synthesis nanomaterials with controllable size and morphology, such as hydrothermal/solvothermal methods,^[^ ^51^, ^52^ ^]^ hot‐injection methods,^[^ ^53^ ^]^ molten synthesis,^[^ ^54^ ^]^ template‐assisted synthesis,^[^ ^55^ ^]^ and chemical/physical vapor deposition.^[^ ^56^, ^57^ ^]^ Different from the conventional nanomaterials, the SNMs have the size in single‐unit cell level; therefore, it is a great challenge for their high‐quality synthesis. In recent years, our group has successfully synthesized many types of SNMs using hydrothermal/solvothermal methods.^[^ ^51^, ^52^, ^58^, ^59^, ^60^, ^61^, ^62^, ^63^, ^64^, ^65^, ^66^, ^67^, ^68^, ^69^, ^70^, ^71^, ^72^ ^]^ For instance, the nickel hydroxylphosphate single‐wall nanotubes were prepared in the mixture solvents of water and DMF,^[^ ^51^ ^]^ and the tungsten oxide nanobelts with thickness of 1 nm were synthesized in the mixture solvents of water, oleylamine, and cyclohexane.^[^ ^52^ ^]^ With the advantages of simple manipulation, controllable adjustment of precursor ratios and high‐purity products, the hydrothermal/solvothermal methods become an efficient means for the universal synthesis of SNMs.

In this section, we will devote our attentions to this hydrothermal/solvothermal strategy. First, we will discuss some examples based on a traditional growth mechanism—the LaMer pathway. After that, we will introduce a new type of growth mechanism—the cluster–nuclei coassembly pathway, which is an attractive approach for the synthesis of SNMs. Finally, we will briefly summarize the differences between these two pathways.

### 2.1 The LaMer Pathway

**Figure 1:**
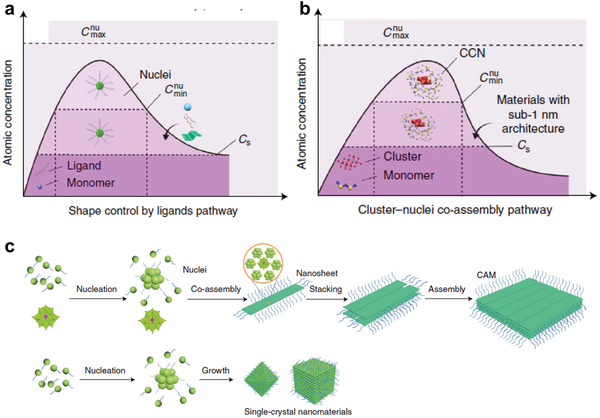
Two typical growth pathways of nanomaterials: a) the LaMer pathway and b) the cluster–nuclei coassembly pathway. c) A comparison of two different growth pathways. Reproduced with permission.^[^ ^59^ ^]^ Copyright 2019, Springer Nature.

LaMer model is a classical growth mechanism of nanomaterials in solution‐phase synthesis.^[^ ^2^ ^]^ In the LaMer pathway, the growth process of nanomaterials can be divided into two stages: a nucleation stage and a growth stage (**Figure** **1**a).^[^ ^59^ ^]^ In the nucleation stage, monomers are interacted with ligands to form nuclei; and in the growth stage, the nuclei will continue to grow into different types of nanomaterials. Typically, our group developed a “good solvent and poor solvent” system for a universal synthesis of SNMs based on the solvothermal method, such as InS nanocoils,^[^ ^60^ ^]^ WO*_x_* nanobelts,^[^ ^52^ ^]^ and GdOOH ultrathin nanowire.^[^ ^23^ ^]^ In this system, good solvent can dissolve the samples, while poor solvents can increase their supersaturation, which can regulate the crystal growth process.^[^ ^20^ ^]^

**Figure 2:**
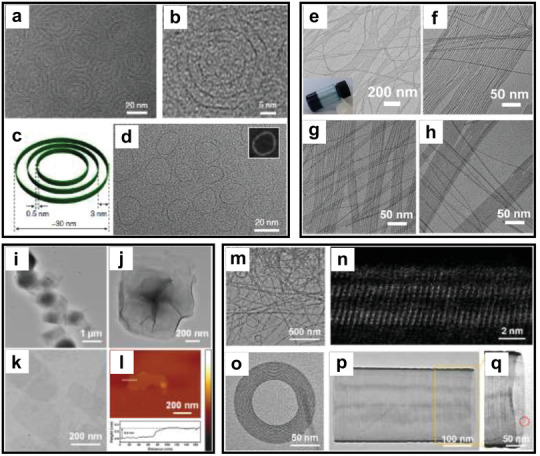
a,b) TEM images of the mSMO NRs. c) The structural model of the mSMO NRs. d) TEM image and HAADF‐STEM image of the sSMO NRs. Reproduced under terms of the CC‐BY license.^[^ ^61^ ^]^ Copyright 2017, The Authors. Published by Springer Nature. e–h) TEM images of the different kinds of QTBNWs. (e,f) Na‐Cs‐QTBNWs, (g) Na‐Li‐QTBNWs, and (h) K‐Cs‐QTBNWs. Reproduced with permission.^[^ ^62^ ^]^ Copyright 2018, American Chemical Society. i,j) TEM images of the MoO*_x_* HNS. k) TEM image of the single‐layer MoO*_x_* HNS. l) AFM image of the single‐layer MoO*_x_* HNS. Reproduced with permission.^[^ ^63^ ^]^ Copyright 2018, Wiley‐VCH. m) TEM image of the MoO_3‐_ *_x_* SNW. n) Atomic‐resolution HAADF‐STEM image of the 1D MoO_3‐_ *_x_* SNW. o) TEM image of the 2D MoO_3‐_ *_x_* SNW. p,q) TEM images of the 3D MoO_3‐_ *_x_* SNW. Reproduced with permission.^[^ ^64^ ^]^ Copyright 2018, American Chemical Society.

In 2017, our group fabricated atomic‐level sulfur‐doped molybdenum oxide nanorings using ammonium heptamolybdate and thiourea as precursors in the mixed solvent of water, oleyl alcohol, and oleylamine (**Figure** **2**a–d).^[^ ^61^ ^]^ The thickness of the nanorings is ≈0.5 nm, and it showed a tunable ring‐in‐ring structure; the diameter of the exterior nanorings was 30 nm, and the ring spacing was ≈3.0 nm. By changing the amounts of thiourea, both multilevel nanorings (mSMO NRs) and single‐level nanorings (sSMO NRs) were obtained. Meanwhile, this multilevel ring structure could also be obtained by selenium doping, and the sSMO NRs could be prepared by tuning the solvent ratio. Later on, quaternary tungsten bronze nanowires (QTBNWs) with diameter of 1–2 nm and length in micrometer scale were obtained by using deionized water as the poor solvent, and cyclohexane and oleylamine as the good solvents, including Na‐Cs‐QTBNWs, Na‐Li‐QTBNWs, and K‐Cs‐QTBNWs (Figure 2e–h).^[^ ^62^ ^]^ In this work, the growth of QTBNWs could be divided into three stages: first, some tungsten oxide clusters were formed and assembled into nanosheets assisting with oleylamine; then, the alkali metal ions entered the nanosheets and further induced the transformation from nanosheets to nanobelts; finally, oleylamine acted as a soft template, guiding the formation of nanowires.

Besides these solvothermal methods, the SNMs can also be synthesized using hydrothermal methods. We successfully prepared oxygen‐defected molybdenum oxides hierarchical nanostructure (MoO*_x_* HNS) possessing flower‐like morphology with an average size of ≈1 µm, which was composed of ultrathin nanosheets with thickness of 0.3 nm.^[^ ^63^ ^]^ In this work, we used glycine as the reducing reagent and water as the solvent (Figure 2i–l). In this hydrothermal process, the organic–inorganic hybrids dissolved and formed molybdenum oxide clusters; after that, the clusters arranged together to form the ultrathin nanosheets, and further assembled to the MoO*_x_* HNS. By changing the ratio of precursors, we also synthesized single‐crystal inorganic helical architectures composed by MoO_3‐_ *_x_* sub‐nanometric wires (MoO_3‐_ *_x_* SNW) (Figure 2m–q).^[^ ^64^ ^]^ The major morphology of the MoO_3‐_ *_x_* SNW was 1D nanobelts with a width of 30–50 nm and length of micrometers. Meanwhile, 2D nanocoils (diameter of 100–200 nm and width of 30–50 nm) and 3D nanotubes (diameter of about 250 nm) with helical architectures could also be observed. The growth process of the MoO_3‐_ *_x_* SNW can be divided into three stages: first, the organic–inorganic hybrids were formed under stirring; then, the hybrids grew up as the reaction proceeds, and the nanowires appeared; finally, the formed hybrids dissolved, and the products were obtained.

### 2.2 The Cluster–Nuclei Coassembly Pathway

**Figure 3:**
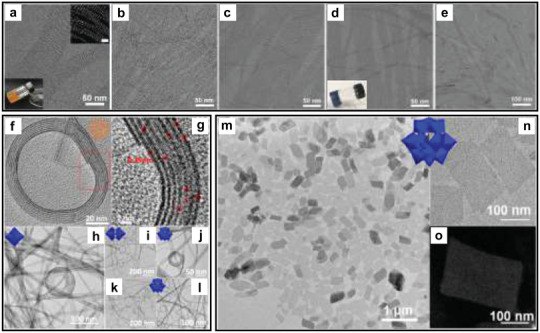
a–e) TEM images of the a) Fe_3_O_4_–PMoO SNWs, b) Y_2_O_3_–PMoO SNWs, c) Yb_2_O_3_–PMoO SNWs, d) Bi_2_O_3_–PMoO SNWs, and e) CeO_2_–PMoO SNWs. Reproduced with permission.^[^ ^67^ ^]^ Copyright 2021, Wiley‐VCH. f,g) Cryoelectron microscopy images of the MoO_3_–W_6_O_19_ HSNSs. h–l) TEM images of the h) MoO_3_–W_6_O_19_ HSNSs, i) MoO_3_–W_10_O_32_ HSNSs, j) MoO_3_–PW_12_O_40_ HSNSs, and k,l) MoO_3_–SiW_12_O_40_ HSNSs. Reproduced with permission.^[^ ^68^ ^]^ Copyright 2020, American Chemical Society. m,n) TEM images of the CuO–PMA SNSs. o) HAADF‐STEM image of the CuO–PMA SNSs. Reproduced with permission.^[^ ^69^ ^]^ Copyright 2019, American Chemical Society.

In 2019, our group first proposed the cluster–nuclei coassembly pathway for the synthesis of SNMs (Figure 1b).^[^ ^59^ ^]^ Clusters, such as POMs, have a close size with inorganic nuclei. At the nucleation stage, clusters will interact with monomers and nuclei, and further grow into various SNMs. Based on the cluster–nuclei coassembly strategy, both Au–PW_12_ sub‐nanowires^[^ ^65^ ^]^ and TiO_2_/ZrO_2_–PMoO sub‐nanobelts^[^ ^66^ ^]^ have been successfully prepared, showing the generality of this strategy for synthesizing SNMs, especially for the hybrid SNMs. By adding phosphomolybdic acid in the synthetic system, we developed a generic method to obtain various kinds of sub‐nanowires (including Fe_3_O_4_–PMoO SNWs, Y_2_O_3_–PMoO SNWs, Yb_2_O_3_–PMoO SNWs, Bi_2_O_3_–PMoO SNWs, and CeO_2_–PMoO SNWs) (**Figure** **3**a–e).^[^ ^67^ ^]^ All kinds of the SNWs possessed a length in micrometer scale and a diameter of sub‐nanometer. According to the investigation of the growth mechanism, both the POM clusters and the good/poor solvent system synergically promote the formation of the SNWs. The POM clusters were first interact with nuclei of metal oxides to form sub‐nanometer building blocks, and further assembled into SNWs in the good/poor solvent system. When the tungsten‐based POM clusters were introduced into the MoO_3_ system, four types of hybrid sub‐1 nm nanobelt superstructures (including MoO_3_–W_6_O_19_ HSNSs, MoO_3_–W_10_O_32_ HSNSs, MoO_3_–PW_12_O_40_ HSNSs, and MoO_3_–SiW_12_O_40_ HSNSs) could be synthesized (Figure 3f–l).^[^ ^68^ ^]^ All these HSNSs showed super‐flexible, and could shrink, bend, curl, and twist randomly. According to the molecular dynamics (MD) simulation results, the introduction of POMs could not only interact with MoO_3_ to form HSNSs, but also lead to the bending of the structures. Except for the 1D sub‐nanowires and sub‐nanobelts, 2D sub‐nanosheets can also be synthesized using this pathway (Figure 3m–o).^[^ ^69^ ^]^ Specifically, phosphomolybdic acid clusters were incorporated into CuO during the nucleation step, and the CuO–PMA sub‐nanosheets (SNSs) were obtained. The shape of the CuO–PMA SNSs was nearly rectangular, with the size of several hundred nanometers and the thickness of about 1 nm. MD simulations could also prove this cluster–nuclei coassembly pathway in this work, in which CuO and PMA would interact at the nucleation stage, and further grew into SNSs.

### 2.3 Comparisons between Both Two Pathways

Here, we will summarize the differences between the LaMer pathway and the cluster–nuclei coassembly pathway. The differences between these two pathways are mainly reflected in the nucleation stage (Figure 1c). In the LaMer pathway, the monomers aggregate into nuclei with the assistance of ligands. Hence, ligands play a decisive role in controlling the size and morphology of the SNMs. Thus, this method can also be called “ligands pathway.” However, the cluster–nuclei coassembly pathway is quite different from the LaMer pathway. In the cluster–nuclei coassembly pathway, the formed nuclei will assemble with clusters, and the morphology of the SNMs is determined by the combined action of clusters and ligands. It should be noted that, the final products prepared using the cluster–nuclei coassembly pathway are not only SNMs, but also other nanomaterials with larger size, such as CoO–POM janus‐like ultrathin nanosheets,^[^ ^70^ ^]^ POM–ZrO_2_ belt like superstructures,^[^ ^71^ ^]^ and CoO–Mo_8_ ultrathin nanowires.^[^ ^72^ ^]^ Therefore, in addition to the introduction of clusters, the fine regulation of the reaction system is also important for the synthesis of SMNs.

## 3 Photothermal Energy Conversion of the SNMs

As we mentioned above, photothermal conversion is an important energy conversion method for the efficient utilization of light energy. In this section, we will discuss the photothermal performance of the SNMs under laser irradiation and solar irradiation, respectively. We will also discuss the photothermal conversion mechanism of the SNMs.

### 3.1 Photothermal Performance of the SNMs under Laser Irradiation

**Figure 4:**
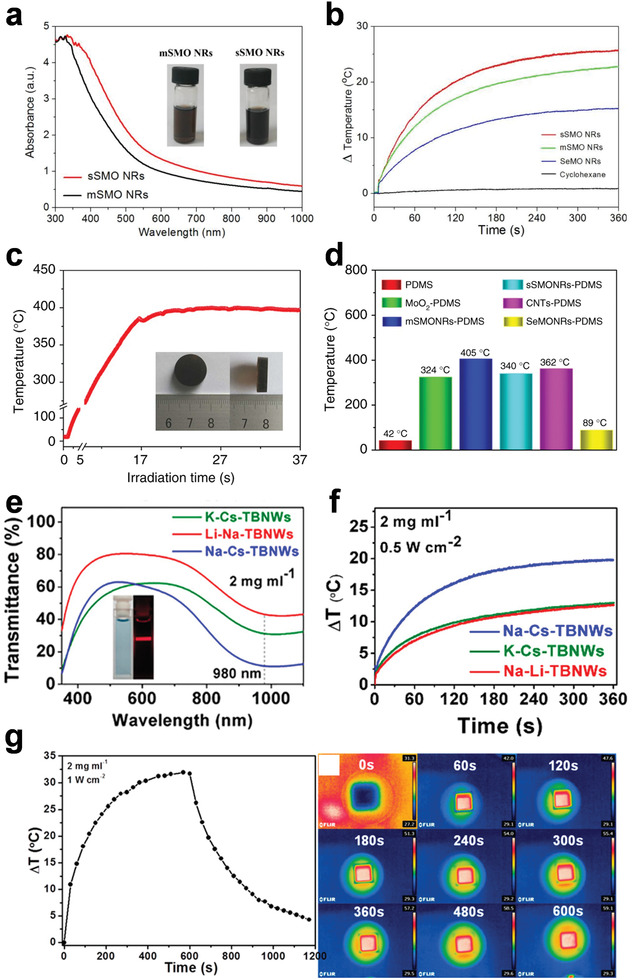
a) UV–vis–NIR absorption spectra of the mSMO NRs and sSMO NRs. b) Photothermal performance of the mSMO NRs, sSMO NRs, and SeMO NRs dispersion under laser irradiation. c,d) Photothermal performance of the sSMONRs–PDMS composite under laser irradiation. Reproduced under terms of the CC‐BY license.^[^ ^61^ ^]^ Copyright 2017, The Authors. Published by Springer Nature. e) Transmittance spectra of the QTBNWs. f) Photothermal performance of the QTBNWs under laser irradiation. Reproduced with permission.^[^ ^62^ ^]^ Copyright 2018, American Chemical Society. g) Photothermal performance of the CuO–PMA SNSs under laser irradiation. Reproduced with permission.^[^ ^69^ ^]^ Copyright 2019, American Chemical Society.

In the previous work by our group,^[^ ^61^ ^]^ the sSMO NRs and mSMO NRs both showed a broad absorption in visible and NIR regions (**Figure** **4**a). With more free electrons due to the more sulfur doping, the sSMO NRs exhibited enhanced light absorption compared with mSMO NRs. As a result, the temperature of the sSMO NRs in cyclohexane solution (0.1 mg mL^−1^) increased by 26 °C with the laser irradiation (808 nm, 1 W cm^−2^) for 6 min, which was much higher than that of the mSMO NRs dispersion (Figure 4b). After introducing the sSMO NRs into polydimethylsiloxane (PDMS), the sSMONRs–PDMS composite showed a unique photothermal performance. Under the laser irradiation (808 nm, 1 W cm^−2^), the local temperature would rise up to 400 °C within 20 s (Figure 4c,d), which is a record temperature induced by light irradiation. This highest temperature could be attributed to the strong light absorption and the excellent compatibility with PDMS of the sSMO NRs, and the thermal accumulation in the PDMS substrate. The QTBNWs also showed good photothermal performance in cyclohexane solutions (Figure 4e,f).^[^ ^62^ ^]^ Among three kinds of QTBNWs samples, the Na‐Cs‐QTBNWs exhibited the best performance compared with the Na‐Li‐QTBNWs and the K‐Cs‐QTBNWs. Under the irradiation of a 980 nm laser (0.5 W cm^−2^), the temperature of the Na‐Cs‐QTBNWs dispersions (2 mg mL^−1^) increased by about 20 °C for 6 min. Compared with the Na‐Cs‐quaternary tungsten bronze nanorods (Na‐Cs‐QTBNRs), the Na‐Cs‐QTBNWs also showed better photothermal performance (the temperature change of the Na‐Cs‐QTBNRs was only 14.5 °C using the same measurement condition), which proving the superiority of the SNMs. As for the CuO–PMA SNSs,^[^ ^69^ ^]^ the temperature of the dispersions in cyclohexane (2 mg mL^−1^) could increase by 31.9 °C within 10 min under an 808 nm laser irradiation with the power intensity of 1 W cm^−2^ (Figure 4g).

**Figure 5:**
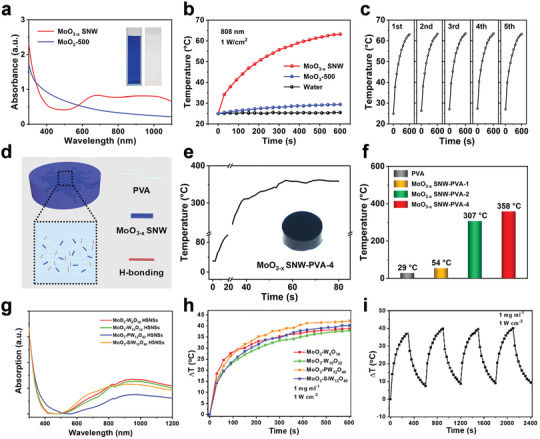
a) UV–vis–NIR absorption spectra of the MoO_3‐_ *_x_* SNW aqueous solution. b,c) Photothermal performance of the MoO_3‐_ *_x_* SNW aqueous solution under laser irradiation. d) Schematic illustration of the MoO_3‐x_ SNW/PVA hydrogel. e,f) Photothermal performance of the MoO_3‐_ *_x_* SNW/PVA hydrogel under laser irradiation. Reproduced with permission.^[^ ^64^ ^]^ Copyright 2018, American Chemical Society. g) UV–vis–NIR absorption spectra of the four kinds of MoO_3_–POM HSNSs. h,i) Photothermal performance of the MoO_3_–POM HSNSs aqueous solution under laser irradiation. Reproduced with permission.^[^ ^68^ ^]^ Copyright 2020, American Chemical Society.

Besides the hydrophobic SNMs, many kinds of hydrophilic SNMs also displayed excellent photothermal performance under laser irradiation. In the case of the MoO_3‐_ *_x_* SNW,^[^ ^64^ ^]^ the absorption spectra showed the enhanced optical activities of the MoO_3‐_ *_x_* SNW, especially in NIR regions (**Figure** **5**a). This MoO_3‐_ *_x_* SNW exhibited excellent photothermal performance in both aqueous solutions and hydrogel matrixes. In the aqueous solution, the temperature of the dispersion (concentration: 1 mg mL^−1^) could rise from 25 to 63 °C under 808 nm laser irradiation (intensity: 1 W cm^−2^) with an energy conversion efficiency of 43.2% (Figure 5b), and there was no attenuation of the performance in a 5‐cycle test (Figure 5c). After introducing the MoO_3‐_ *_x_* SNW into a poly(vinyl alcohol) (PVA) matrix, the local temperature of the MoO_3‐_ *_x_* SNW/PVA hydrogels could reach 358 °C within 80 s (Figure 5d–f). Similar with the sSMONRs–PDMS example mentioned above, this extremely high temperature of the hydrogel composites was induced by the strong light absorption of the MoO_3‐_ *_x_* SNW and the energy accumulation effect of the hydrogel matrix. In the hybrid MoO_3_–POM structures,^[^ ^68^ ^]^ all the four kinds of MoO_3_–POM HSNSs showed strong NIR light absorption and highly efficient photothermal conversion (Figure 5g–i). Among them, the MoO_3_–PW_12_O_40_ HSNSs exhibited the best performance under an 808 nm laser irradiation, with the temperature increase of 42.3 °C within 600 s.

Otherwise, various kinds of 2D nanomaterials with the thickness below 1 nm, which can be regarded as 2D SNMs, have also been applied for the photothermal conversion. Dai and co‐workers first synthesized graphene oxide (GO) sheets and used amine‐terminated poly (ethylene glycol) (PEG) to prepare nanosized GO; after reducing GO by using hydrazine monohydrate and coating it with the polymer, the nanosized reduced graphene oxide (nano‐rGO) sheets with the size from 5 to 100 nm were finally obtained.^[^ ^73^ ^]^ The nano‐rGO sheets showed sixfold higher NIR absorption than the nano‐GO, and the temperature of nano‐rGO (concentration: 20 mg L^−1^) could reach 50 °C within 5 min under an 808 nm laser irradiation (power density: 0.6 W cm^−2^). Zhang and co‐workers reported that single‐layered BP nanosheets could be prepared by liquid exfoliation techniques.^[^ ^74^ ^]^ Under a laser irradiation (power density: 1 W cm^−2^) for 10 min, the temperature of the BP nanosheets solution would increase to about 45 °C. Shi and co‐workers fabricated the Ti_3_C_2_ nanosheets using a two‐step exfoliation strategy, in which the Ti_3_AlC_2_ was first synthesized following the combination of HF etching and TPAOH intercalation.^[^ ^75^ ^]^ Under the irradiation of an 808 nm laser at a power intensity of 1.5 W cm^−2^, the temperature of the Ti_3_C_2_ solution could reach 57 °C in 6 min even at a low concentration (72 ppm). Liu and co‐workers developed an exfoliation method with assistant of Li ion insertion to synthesis single‐layered WS_2_ nanosheets,^[^ ^76^ ^]^ and the solution temperature (concentration: 0.5 mg mL^−1^) would increase more than 60 °C in 5 min under an 808 nm NIR laser irradiation at a power density of 0.8 W cm^−2^. Wei and co‐workers synthesized CuFe–LDH nsnosheets with the thickness of ≈1.3 nm using a “bottom‐up” method, and loaded glucose oxidase onto the nanosheets to build a multifunctional nanosystem (GOD/CuFe–LDH).^[^ ^77^ ^]^ In a weak acid environment, the surface of the CuFe–LDHs would be etched, and the photogenerated electron–hole pairs increased due to defects caused by etching. As a result, the GOD/CuFe–LDH exhibited acid‐enhanced photothermal performance. Under an 808 nm laser irradiation for 10 min (power density: 1 W cm^−2^), the temperature of the solutions (0.1 mg mL^−1^) at pH = 7.4, 6.5, and 5.4 could reach at 44.3, 54.2, and 56.5 °C, respectively, and the corresponding photothermal conversion efficiency was 46.0%, 75.1%, and 83.2%.

### 3.2 Photothermal Performance of the SNMs under Solar Irradiation

**Figure 6:**
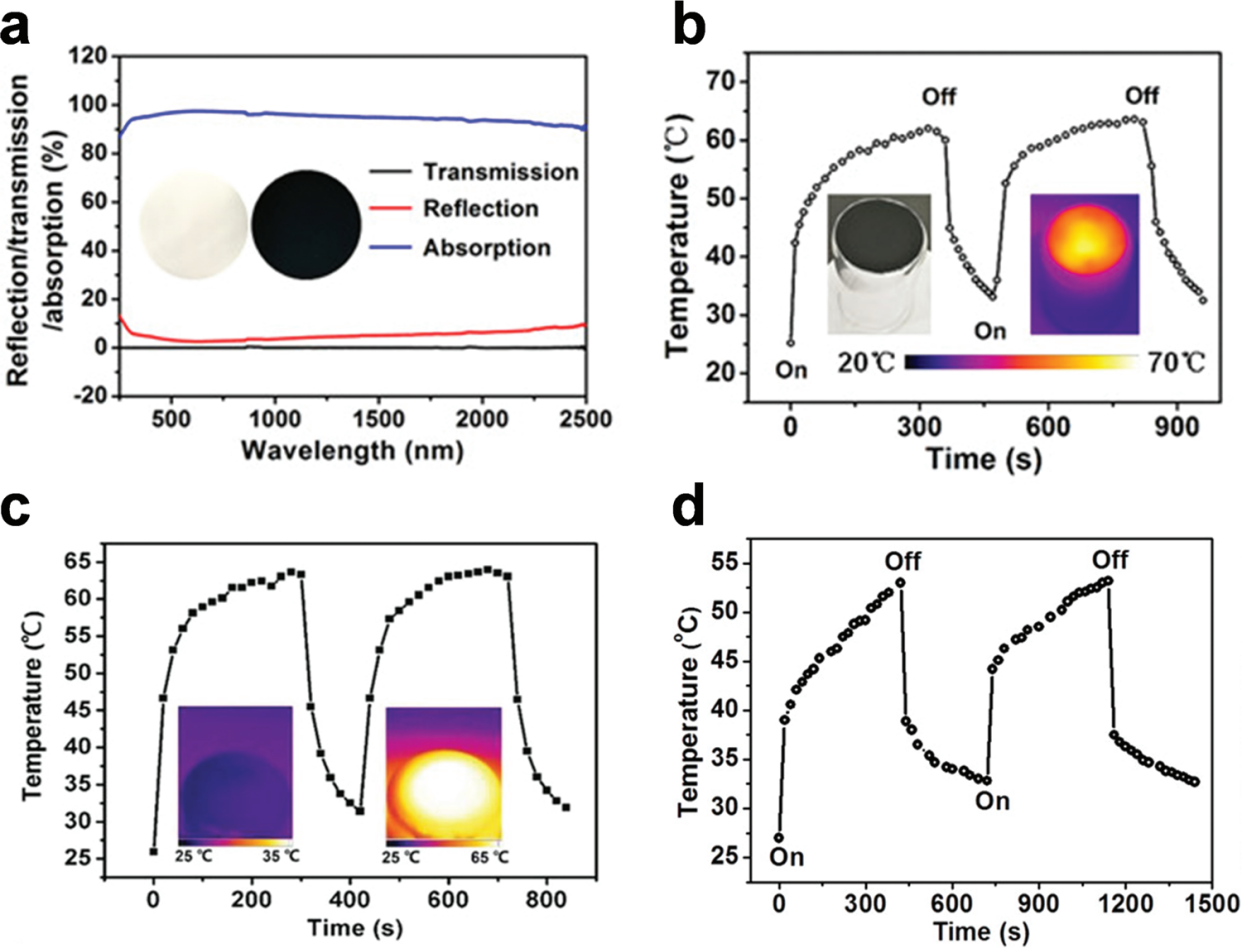
a) Optical properties of the MoO*_x_* HNS membrane. b) Photothermal performance of the MoO*_x_* HNS membrane under solar irradiation. Reproduced with permission.^[^ ^63^ ^]^ Copyright 2018, Wiley‐VCH. c) Photothermal performance of the Bi_2_O_3_–PMoO SNW film under solar irradiation. Reproduced with permission.^[^ ^67^ ^]^ Copyright 2021, Wiley‐VCH. d) Photothermal performance of the CuO–PMA SNSs membrane under solar irradiation. Reproduced with permission.^[^ ^69^ ^]^ Copyright 2019, American Chemical Society.

Different from using laser as the light source, high‐efficient solar energy conversion demands that light absorption range of the photothermal materials need to be expanded to the whole solar spectrum. As a typical example, the MoO*_x_* HNS exhibited strong light absorption ranging from 250 to 2500 nm.^[^ ^63^ ^]^ On the one hand, the oxygen defects enhanced the light absorption of the MoO*_x_* HNS, which will be discussed in detail in the next part. On the other hand, the assembled structure of the MoO*_x_* HNS could reduce the light reflection, and further improved the light absorption compared with the single‐layer MoO*_x_* nanosheets. After loaded on a polytetrafluoroethylene film, the MoO*_x_* HNS membrane showed average absorption of 97% in the visible light range and over 90% absorption in the whole solar spectrum range (**Figure** **6**a). Under the solar irradiation of 1 kW m^−2^ (1 sun), the temperature of the MoO*_x_* HNS membrane could rise from 25 to 62 °C in 6 min, revealing its rapid photothermal response (Figure 6b). Using a wet‐spinning method, the Bi_2_O_3_–PMoO SNWs could be fabricated into films.^[^ ^67^ ^]^ The light absorption of the Bi_2_O_3_–PMoO SNW film was higher than 90% in the range of 250–1560 nm and exceed 80% in the range of 1560–2500 nm. As a result, under 1 sun irradiation, the temperature of the Bi_2_O_3_–PMoO SNW film could rise from 25 to 64 °C in 5 min (Figure 6c). Without the size in sub‐nanometer scale, the Bi_2_O_3_–PMoO nanorods showed poor photothermal performance, the temperature of which could only increase 14.6 °C in 6 min under 1 sun. By loading on the cellulose acetate film, the CuO–PMA SNSs membrane also showed good solar‐to‐thermal conversion performance.^[^ ^69^ ^]^ At 1 sun illumination, the temperature of the membrane could rise from 27 to 53 °C in 7 min with the assistance of the CuO–PMA SNSs (Figure 6d). The synthesis and photothermal performance of the SNMs are summarized in **Table** **1**.

**Table 1:** Summary of the synthesis and photothermal performance of the SNMs

Materials	Morphology	Synthetic method	Light source	Concentration	Performance	Efficiency	Ref.
sSMO NRs	Nanoring (diameter: 30 nm, thickness: 0.5 nm)	Solvothermal method	Laser (808 nm, 1 W cm^−2^)	0.1 mg mL^−1^	Δ*T* = 26 °C for 6 min	–	^[^ ^61^ ^]^
Na‐Cs‐QTBNWs	Nanowire (diameter: 1–2 nm, length: micrometer scale)	Solvothermal method	Laser (980 nm, 0.5 W cm^−2^)	2 mg mL^−1^	Δ*T* = 20 °C for 6 min	–	^[^ ^62^ ^]^
MoO_3‐_ *_x_* SNW	Nanowire assembly (diameter: 1 nm	Hydrothermal method	Laser (808 nm, 1 W cm^−2^)	1 mg mL^−1^	Δ*T* = 38 °C within 10 min	43.2%	^[^ ^64^ ^]^
MoO_3_−PW_12_O_40_ HSNSs	Nanobelt (thickness: 1 nm)	Solvothermal method	Laser (808 nm, 1 W cm^−2^)	1 mg mL^−1^	Δ*T* = 42.3 °C within 10 min	–	^[^ ^68^ ^]^
Nano‐rGO sheets	Nanosheet (single layer, diameter: 20 nm)	Sonication and reduction	Laser (808 nm, 0.6 W cm^−2^)	20 µg mL^−1^	Δ*T* = 25 °C within 5 min	–	^[^ ^73^ ^]^
BP nanosheets	Nanosheet (single layer, diameter: 205.4 ± 5.11 nm)	Liquid exfoliation method	Laser (808 nm, 1 W cm^−2^)	1 mg mL^−1^	Δ*T* = 20 °C within 10 min	–	^[^ ^74^ ^]^
Ti_3_C_2_ nanosheets	Nanosheet (single layer, size: 150 nm)	Chemical exfoliation method	Laser (808 nm, 1.5 W cm^−2^)	72 µg mL^−1^	Δ*T* = 25 °C within 6 min	30.6%	^[^ ^75^ ^]^
WS_2_ nanosheets	Nanosheet (thickness: 1.1 nm, diameter: 50–100 nm)	Li ion insertion method	laser (808 nm, 0.8 W cm^−2^)	0.5 mg mL^−1^	Δ*T* = 60 °C within 5 min	–	^[^ ^76^ ^]^
CuFe–LDH nsnosheets	Nanosheet (thickness: 1.3 nm, diameter: 65 nm)	Bottom‐up method	Laser (808 nm, 1 W cm^−2^)	0.1 mg mL^−1^	Δ*T* = 26.6 °C within 5 min	83.2%	^[^ ^77^ ^]^
CuO–PMA SNSs	Nanosheet (thickness: 1 nm, size: hundreds of nanometers)	Solvothermal method	Laser (808 nm, 1 W cm^−2^)	2 mg mL^−1^	Δ*T* = 31.9 °C within 10 min	–	^[^ ^69^ ^]^
			Solar (1 kW m^−2^)	Solid	Δ*T* = 26 °C within 7 min	–	
MoO*_x_* HNS	Nanosheet assembly (thickness: 0.3 nm)	Hydrothermal method	Solar (1 kW m^−2^)	Solid	Δ*T* = 37 °C within 6 min	–	^[^ ^63^ ^]^
Bi_2_O_3_–PMoO SNWs	Nanowire (diameter: 1 nm, length: micrometer scale)	Solvothermal m method	Solar (1 kW m^−2^)	Solid	Δ*T* = 39 °C within 6 min	–	^[^ ^67^ ^]^

### 3.3 Photothermal Conversion Mechanisms of the SNMs

**Figure 7:**
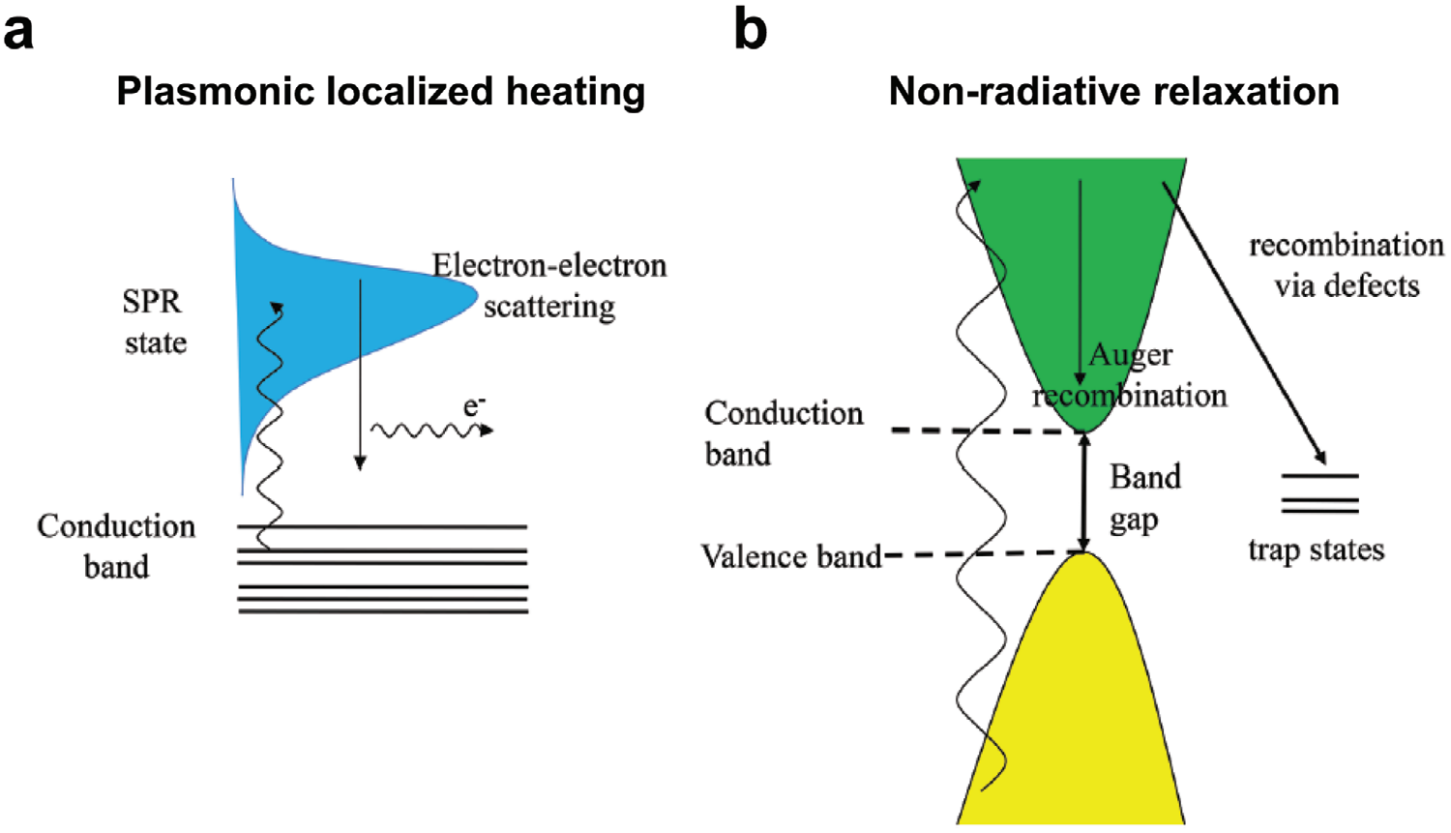
Photothermal conversion mechanisms for light‐absorption materials: a) plasmonic localized heating and b) nonradiative relaxation. Reproduced under terms of the CC‐BY license.^[^ ^78^ ^]^ Copyright 2020, The Authors. Published by Wiley‐VCH.

There are two kinds of photothermal conversion mechanisms for light‐absorption materials: plasmonic localized heating and nonradiative relaxation (**Figure** **7**).^[^ ^78^ ^]^ Plasmonic localized heating usually occurs in noble metal nanomaterials due to their localized surface plasmon resonance (LSPR) effect.^[^ ^18^ ^]^ When light is incident on noble metals materials, if the incident photon frequency is well matched with the inherent frequency of conduction electrons, the nanomaterials will strongly absorb the photon energy and eventually convert it into heat energy. Similar with the noble metal materials, the LSPR effect also exists in some semiconductors, such as MoO_3‐_ *_x_*,^[^ ^79^ ^]^ M_0.33_WO_3_,^[^ ^80^ ^]^ and TiO_2‐_ *_x_*.^[^ ^81^ ^]^ By introducing defects, the free electron density will increase, and further generate the LSPR effect. In the field of SNMs, the photothermal conversion behaviors of the QTBNWs,^[^ ^62^ ^]^ the CuO–PMA SNSs,^[^ ^69^ ^]^ the MoO_3‐_ *_x_* SNW,^[^ ^64^ ^]^ the MoO_3_–POM HSNSs,^[^ ^68^ ^]^ and the Bi_2_O_3_–PMoO SNWs^[^ ^67^ ^]^ can all be ascribed to this mechanism. Compared with traditional nanomaterials, SNMs will have higher free electron density after introducing defects due to their small size, which leads to a stronger LSPR effect.

Nonradiative relaxation is the other photothermal conversion mechanism, especially for semiconductors.^[^ ^82^, ^83^ ^]^ When the energy of incident photons is higher than the bandgap energy of semiconductors, valance electrons will be excited into conduction band, following by falling back to lower energy bands. During this process, the heat will be generated after the phonon interacts with the lattice, defects, or surface dangling bonds. For example, in the case of MoO*_x_* HNS,^[^ ^63^ ^]^ the defect energy levels would appear after introducing oxygen defects, which induced a significant enhancement of the light absorption. Considering that SNMs have a higher ratio of surface atoms, they will show a more efficient photothermal conversion compared with traditional nanomaterials.

Based on the mentioned mechanisms, the photothermal performance of SNMs can be optimized in two aspects: the first one is tuning the size of the SNMs, and the other one is increasing the ratio of defects or doping in the SNMs. It also should be noted that, in some cases (e.g., transition metal oxides with oxygen defects), both two mechanisms can explain the photothermal conversion process. Hence, more in‐depth researches on photothermal conversion process are demanded, and more sufficient evidences need to be provided to investigate the effect of different mechanisms in photothermal conversion process.

## 4 Applications of SNMs in Photothermal Conversion

Based on the excellent photothermal conversion performance, SNMs display great application potential in various fields, including solar vapor generation, biomedicine, and light‐responsive composites construction (**Table** **2**).

**Table 2:** Summary of the applications of photothermal SNMs

Applications	Materials	Performance	Ref.	Shortcoming
Solar vapor generation	MoO*_x_* HNS	Evaporation rate: 1.255 kg m^−2^ h^−1^, efficiency: 85.6% (1 sun)	^[^ ^63^ ^]^	SNMs may agglomerate during long‐term operation, resulting in the performance attenuation
	CuO–PMA SNSs	Evaporation rate: 1.41 kg m^−2^ h^−1^, efficiency: 95.72% (1 sun)	^[^ ^69^ ^]^	
	Bi_2_O_3_–PMoO SNW	Evaporation rate: 1.38 kg m^−2^ h^−1^, efficiency: 91.1% (1 sun)	^[^ ^67^ ^]^	
Biomedicine	PEG@S‐MoO*_x_* A‐NRs	Synergistic PDT/PTT effect; PA imaging	^[^ ^93^ ^]^	To ensure the biocompatibility of SNMs, surface modification is necessary, which may decrease their photothermal properties
	Nb_2_C‐PVP	Biodegradable; PTT in both the NIR‐I and NIR‐II biowindows	^[^ ^94^ ^]^	
	MnO_2_ nanosheets	PTT; PA imaging	^[^ ^95^ ^]^	
	Ti_3_C_2_‐SP	Drug‐delivery; synergistic PTT and chemotherapy of cancer	^[^ ^96^ ^]^	
Light‐responsive composites construction	sSMO NRs	Light‐responsive shape memory behaviors; self‐healing performance; reversible actuation and dynamic 3D changes	^[^ ^61^ ^]^	Some SNMs have dispersion problems in polymer matrixes
	MoO_3‐_ *_x_* SNW	Light‐responsive shape Memory behaviors	^[^ ^64^ ^]^	

### 4.1 Solar Vapor Generation

The scarcity of freshwater is a severe global problem in the last few decades, and interfacial solar vapor generation (ISVG) is one of the efficient approaches to solving this problem.^[^ ^84^ ^]^ Different from volumetric solar vapor generation (VSVG), the generated heat in the ISVG system can be localized at the air–water interface, which substantially reduces the heat losses to bulk water, and achieves high‐efficient energy conversion. Meanwhile, the cost of ISVG is much lower than that of VSVG, which can meet the requirement of the actual industrial production.^[^ ^85^ ^]^ Up to now, many kinds of light‐absorbing materials have been applied in the field of ISVG, including metallic materials,^[^ ^57^, ^86^ ^]^ semiconductors,^[^ ^87^, ^88^ ^]^ carbon‐based materials,^[^ ^89^, ^90^ ^]^ and polymers.^[^ ^91^, ^92^ ^]^ Among all these photothermal materials, the SNMs with a broad light absorption in the whole solar spectral range show rapid vapor generation rate and high energy conversion efficiency.^[^ ^67^, ^69^, ^73^ ^]^

**Figure 8:**
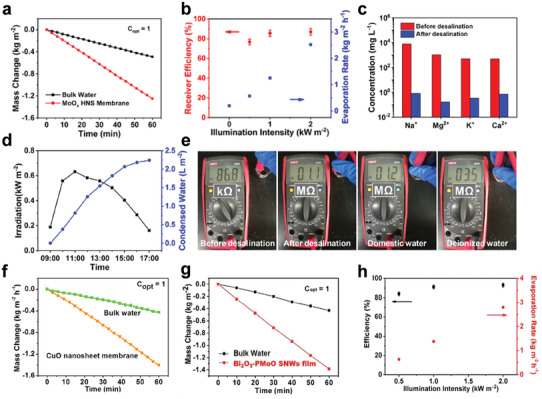
a) SVG performance of the MoO*_x_* HNS membrane under 1 sun irradiation. b) The SVG rate and corresponding efficiency of the MoO*_x_* HNS membrane under different irradiation intensities. c) The concentration of four types of ions in seawater before and after desalination under simulated sunlight irradiation. e) The resistance test of water before and after desalination under natural sunlight irradiation. Reproduced with permission.^[^ ^63^ ^]^ Copyright 2018, Wiley‐VCH. f) SVG performance of the CuO–PMA SNSs membrane under 1 sun irradiation. Reproduced with permission.^[^ ^69^ ^]^ Copyright 2019, American Chemical Society. g) SVG performance of the Bi_2_O_3_–PMoO SNW film under 1 sun irradiation. h) The SVG rate and corresponding efficiency of the Bi_2_O_3_–PMoO SNW film under different irradiation intensities. Reproduced with permission.^[^ ^67^ ^]^ Copyright 2021, Wiley‐VCH.

The MoO*_x_* HNS membrane with an expandable polyethylene foam was applied in the ISVG setup to further reduce heat losses,^[^ ^63^ ^]^ and the temperature nearby the membrane was much higher than that of bulk water under solar irradiation. At 1 sun, the water evaporation rate of the setup with the assistance of MoO*_x_* HNS membrane was 1.255 kg m^−2^ h^−1^, which was 2.9 times of bulk water, and the corresponding energy efficiency was 85.6% (**Figure** **8**a,b). Meanwhile, the MoO*_x_* HNS membrane showed excellent seawater desalination capability under both simulated and natural sunlight irradiation. After treatment, the concentration of four types of ions (Na^+^, Mg^2+^, K^+^, Ca^2+^) in water were decreased by 3–4 orders (Figure 8c), and the resistance value could increase to the value of domestic water (Figure 8e), both of which revealed a high efficiency of desalination process. The CuO–PMA SNSs membrane could also be used in the ISVG setup,^[^ ^69^ ^]^ and it can achieve a high water evaporation rate of 1.41 kg m^−2^ h^−1^ with an efficiency of 95.72% under 1 sun illumination (Figure 8f). Different from both two mentioned examples, the Bi_2_O_3_–PMoO SNW could be processed into a film without any supporters, and it could float on the water surface directly.^[^ ^67^ ^]^ After thermal management, the Bi_2_O_3_–PMoO SNW film could enhance the water evaporation rate from 0.43 to 1.38 kg m^−2^ h^−1^ compared with bulk water under 1 sun illumination, and the corresponding energy efficiency was calculated to be 91.1% (Figure 8g,h).

### 4.2 Biomedicine

**Figure 9:**
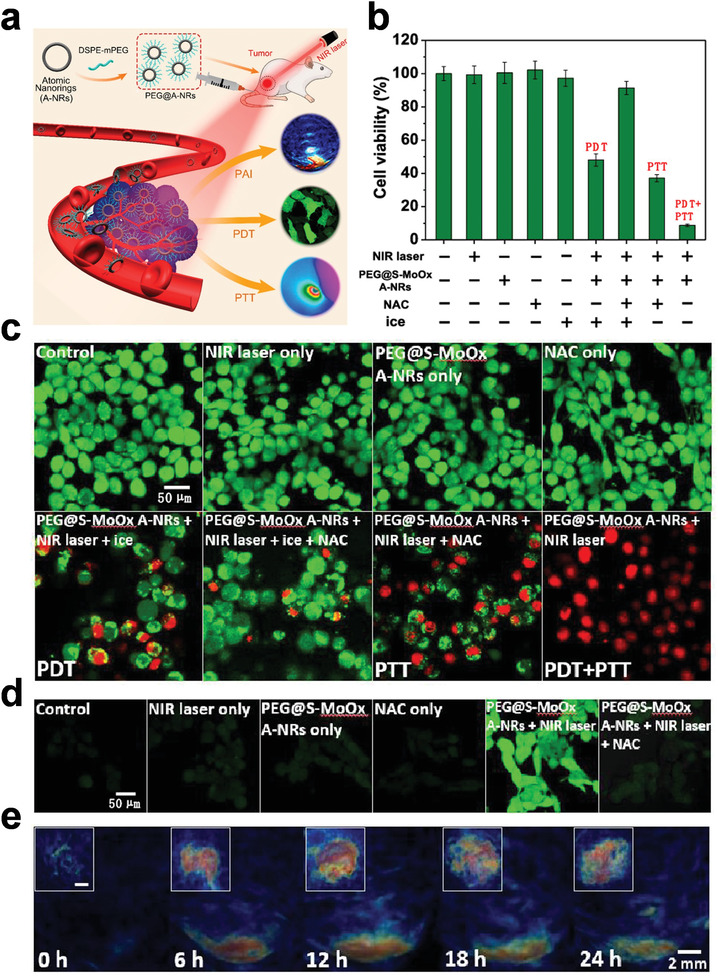
a) Schematic illustration of the S‐MoO*_x_* A‐NRs for PA imaging, PDT, and PTT in the field of cancer therapy. b) Cell viabilities and c) confocal fluorescence images in different treatment groups. d) Images for detecting cellular ROS production in 4T1 cells after different treatments. e) PA images of the tumor tissue in a mouse. Reproduced with permission.^[^ ^93^ ^]^ Copyright 2019, American Chemical Society.

SNMs are also widely applied in the biomedical field, such as PTT,^[^ ^93^, ^94^, ^95^, ^96^ ^]^ PA imaging,^[^ ^93^, ^95^ ^]^ and drug delivery.^[^ ^96^ ^]^ We coated the polyethylene glycol on the surface of atomic‐level sulfur‐doped molybdenum oxide nanorings (PEG@S‐MoO*_x_* A‐NRs) to improve its biocompatibility, which showed low cytotoxicity in vitro.^[^ ^93^ ^]^ The modified sample could be used as a PA imaging‐guided contrast agent, as well as allow PTT/photodynamic therapy (PDT) combinational cancer therapy (**Figure** **9**a). After modification, the PEG@S‐MoO*_x_* A‐NRs also showed high photothermal conversion efficiency. Meanwhile, it could produce reactive oxygen species (ROS) during NIR irradiation. As a result, the synergistic PDT/PTT effect could kill over 90% of the cancer cells, while the PDT effect only and the PTT effect only resulted in 48.08 ± 3.67% and 8.9 ± 2.13% cell survival, respectively (Figure 9b–d). After injecting the PEG@S‐MoO*_x_* A‐NRs into the breast cancer BALB/c model mice, the sample could accumulate at the tumor tissue according to the PA images (Figure 9e), and exhibit excellent in vivo therapeutic effect under laser irradiation. Meanwhile, the PEG@S‐MoO*_x_* A‐NRs would be cleared by macrophage in reticuloendothelial systems, and the half‐life in the blood was 1.56 h, which could ensure an effective accumulation at the tumor area.

**Figure 10:**
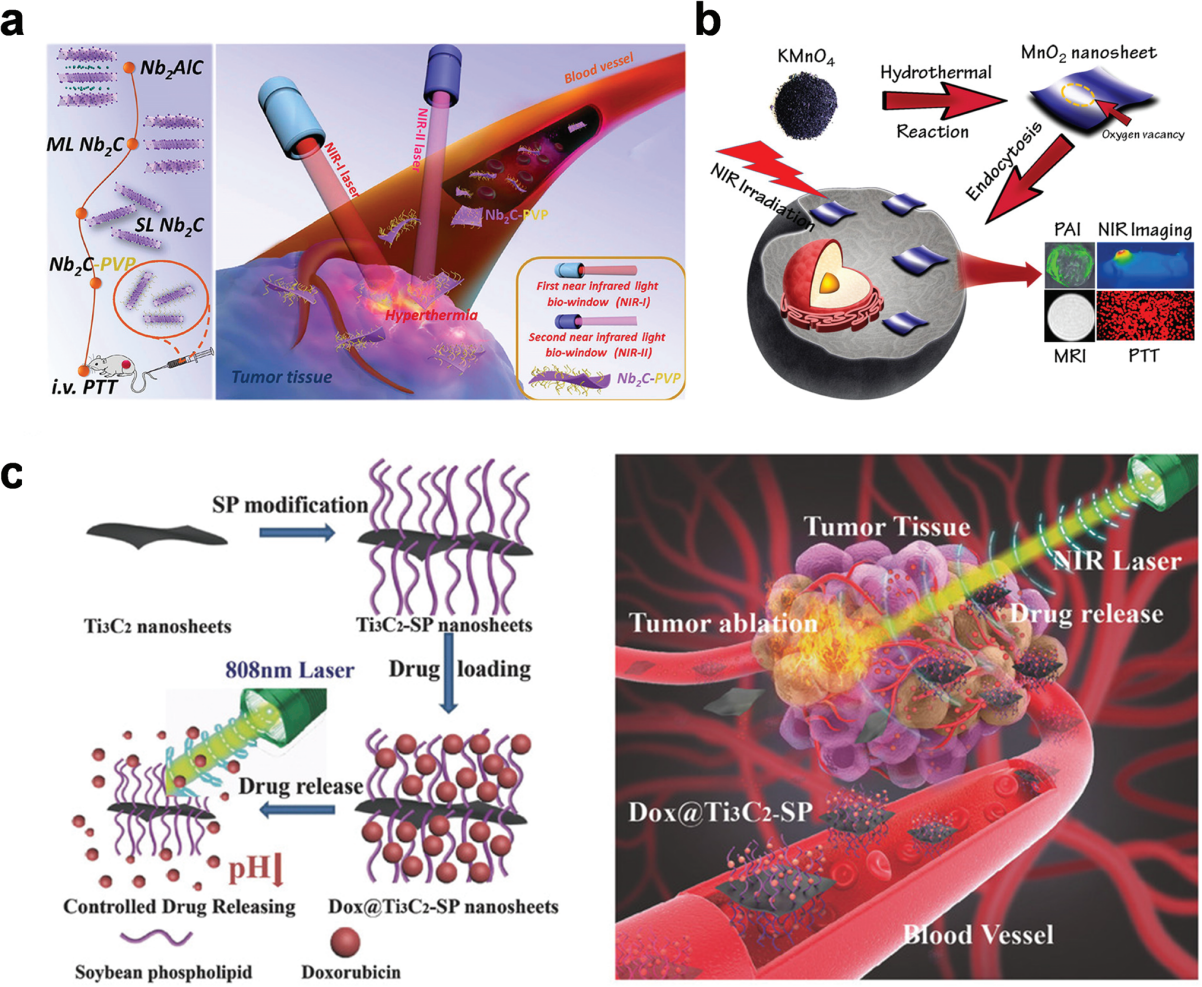
a) Schematic illustration of the 2D Nb_2_C for photothermal tumor ablation in NIR‐I and NIR‐II Biowindows. Reproduced with permission.^[^ ^94^ ^]^ Copyright 2017, American Chemical Society. b) Schematic illustration of the ultrathin MnO_2_ nanosheets for imaging‐guided cancer therapy. Reproduced with permission.^[^ ^95^ ^]^ Copyright 2019, American Chemical Society. c) Schematic illustration of the 2D Ti_3_C_2_ drug‐delivery system for the synergistic PTT and chemotherapy of cancer. Reproduced with permission.^[^ ^96^ ^]^ Copyright 2018, Wiley‐VCH.

Shi and co‐workers applied the 2D niobium carbide (Nb_2_C) MXene for photothermal tumor ablation in both the NIR‐I (750–1000 nm) and NIR‐II biowindows (1000–1350 nm).^[^ ^94^ ^]^ After modified with PVP, the Nb_2_C‐PVP showed excellent biocompatibility without marked toxicity. Notably, it was biodegradable with human myeloperoxidase, which provides a potential harmless route for in vivo applications (**Figure** **10**a). Zhou and co‐workers reported ultrathin MnO_2_ nanosheets with the thickness of 1.2 nm.^[^ ^95^ ^]^ In the in vitro results, the obtained MnO_2_ showed good therapy efficacy to cancer cells. Moreover, in the in vivo tests, it could accumulate in the tumor sites after 3 h injection and exhibit strong PA signal, which lead to the excellent behaviors of the MnO_2_ for imaging‐guided cancer therapy (Figure 10b). Chen and co‐workers reported 2D Ti_3_C_2_ MXene nanosheets with the thickness of about 0.9 nm.^[^ ^96^ ^]^ After modified with soybean phospholipid, the Ti_3_C_2_‐SP could be used as the drug‐delivery system due to its ultrathin planar structure and large surface area. The amount of Dox drug loading of Ti_3_C_2_‐SP was 211.8%, and the Dox releasing ratio could reach 58.0% in the acidic microenvironment. Under NIR laser irradiation, the Ti_3_C_2_‐SP exhibited high photothermal conversion efficiency and enhanced Dox releasing performance. Hence, the Ti_3_C_2_‐SP was employed for the synergistic PTT and chemotherapy of cancer (Figure 10c). It should be noted that, biosafety is an important issue for the biomedical application, especially for the SNMs which have small size. Therefore, the biodegradation behavior of SNMs in the in vivo conditions needs as many attentions as their cancer therapy performance in the future research.

### 4.3 Light‐Responsive Composites Construction

**Figure 11:**
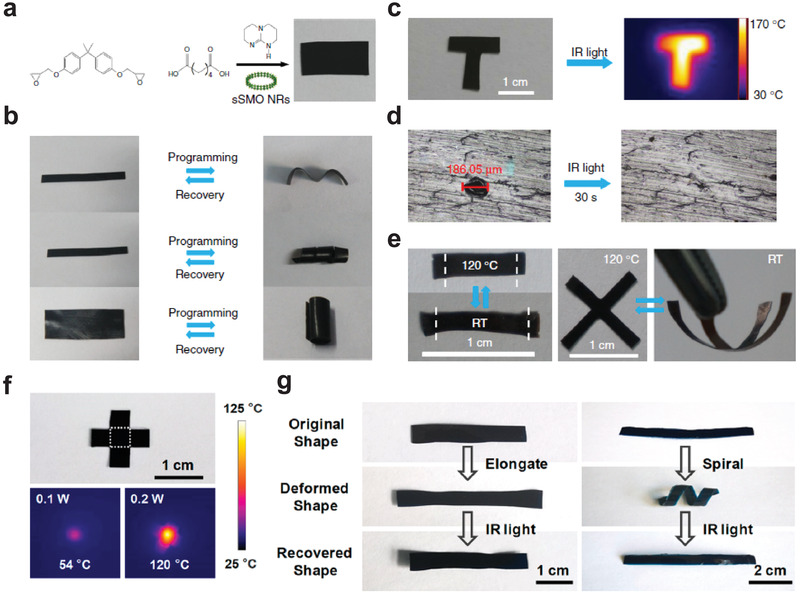
a) Synthesis route of the sSMONRs–vitrimer. b) Shape memory behaviors of the sSMONRs–vitrimer. c) Photothermal image of the sSMONRs–vitrimer under laser irradiation. d) Self‐healing performance of the sSMONRs–vitrimer. e) Reversible actuation and dynamic 3D changes of the sSMONRs–xLCEs. Reproduced under terms of the CC‐BY license.^[^ ^61^ ^]^ Copyright 2017, The Authors. Published by Springer Nature. f) Photothermal image of the MoO_3‐_ *_x_* SNW–PVA film under laser irradiation. g) Shape memory process of the MoO_3‐_ *_x_* SNW–PVA film. Reproduced with permission.^[^ ^64^ ^]^ Copyright 2018, American Chemical Society.

When introducing photothermal SNMs into the thermoresponsive functional polymers, the temperature of the composites will rise up under light irradiation, and the light‐response behaviors of the composites can be achieved. On the one hand, the SNMs can be well dispersed in the polymer matrix because of their small size and controllable surface hydrophilicity–hydrophobicity. On the other hand, the temperature of composites is tunable by adjusting the concentration of SNMs and the intensity of light irradiations, and the manipulation of light‐responsive composites can be precisely controlled by changing their temperature. We introduced the sSMO NRs into a thermoresponsive vitrimer (**Figure** **11**a), and investigated the light‐responsive shape memory effects and self‐healing performance of the composites.^[^ ^61^ ^]^ For the shape memory effects, the sSMONRs–vitrimer strip was first reconfigured into various temporary geometries. Under visible‐light irradiation (Intensity: 140 mW cm^−2^), the temperature of the composites could increase over its glass‐transition temperature, and these temporary geometries would recover to the original shape in seconds (Figure 11b). For the self‐healing performance, NIR laser irradiation was applied, and the damaged area in the sSMONRs–vitrimer would self‐heal when the temperature increased over its topology‐freezing transition temperature (*T*_v_) (Figure 11c,d). Liquid crystalline elastomers with exchangeable links (xLCEs) were further selected as polymer substrates to expand the scope of light‐responsive functional composites. Under laser irradiation, the sSMONRs–xLCEs could achieve reversible actuation and dynamic 3D changes (Figure 11e). We also tried to introduce SNMs into hydrogel matrixes to fabricate light‐responsive functional composites. As a typical example, the MoO_3‐_ *_x_* SNW–PVA film could achieve shape memory behaviors under NIR laser irradiation (Figure 11f,g).^[^ ^64^ ^]^

## 5 Conclusion

In this review, we have discussed the SNMs and their synthetic methods. Considering the single‐unit‐cell size of SNMs, a hydrothermal/solvothermal strategy was developed for the general synthesis of SNMs. There are two growth mechanisms in this synthesis strategy: i) the LaMer pathway, which is a traditional mechanism. In this pathway, nuclei are formed by the interaction of monomers and ligands, and then grow into SNMs. ii) the cluster–nuclei coassembly pathway, which is a new type of mechanism. In this pathway, clusters will interact with monomers and nuclei at the nucleation stage. Because the composition of clusters can be different from monomers, this pathway can be used for the general synthesis of hybrid SNMs. We have also summarized the photothermal conversion performance of the SNMs. As an efficient energy conversion approach, photo‐to‐thermal conversion based on SNMs can be achieved under light irradiations. In the case of the SNMs with enhanced NIR light absorption, the temperature of solutions can rise up rapidly under a NIR laser irradiation. Meanwhile, using a polymer as a substrate, the local temperature can even reach 350–400 °C due to the thermal accumulation of the polymer matrixes. As for the SNMs with strong absorption in the whole solar spectrum, the solar energy can be converted into heat energy efficiently. We analyzed the photothermal conversion mechanisms of the different kinds of SNMs. There are two main mechanisms for the photothermal conversion: i) plasmonic localized heating induced by LSPR effect of the SNMs, ii) nonradiative relaxation. Notably, in some particular cases, such as transition metal oxides with oxygen defects, both two mechanisms are existed in the photothermal conversion process, which need to be more in‐depth studied in future. Due to the excellent photothermal performance, the SNMs can be applied in various fields, such as solar vapor generation, biomedicine, and light stimulus response composites. In the ISVG system, the light‐absorbed SNMs can achieve a rapid water evaporation with a high‐efficient solar‐to‐vapor conversion. As for biomedicine, the SNMs can be used for cancer photothermal therapy, photoacoustic imaging, and drug delivery. Meanwhile, the SNMs can be introduced into polymer matrixes to achieve light‐response behaviors under laser irradiations, such as shape memory effects and self‐healing performance, as well as reversible actuations and dynamic 3D changes.

In summary, SNMs with strong light absorption can be used in the field of photothermal energy conversion. However, there are still many challenges in this field. First, the synthesis of SNMs needs to be further developed, especially for the functional inorganic compositions. Due to the restriction of the synthetic technology, the variety of SNMs is very limited. Although some new strategies have been developed for the universal synthesis of SNMs (such as the hydrothermal/solvothermal approach, and the cluster–nuclei coassembly pathway), the parameters of SNMs (including composite, shape, size, etc.) have not been easily regulated as expect yet. Hence, further development of the synthetic methods is a very important research topic. Second, the photothermal conversion mechanisms of the SNMs should be deeply explored, which can facilitate for the structure design of SNMs with enhanced photothermal performance. Due to the small size, the SNMs are advantageous over the traditional nanomaterials in the field of photothermal energy conversion. The SNMs have high ratio of surface atoms, and the light–matter interaction in SNMs will be stronger than that in traditional nanomaterials. Thus, to further enhance the photothermal efficiency of SNMs, reducing the size and increasing the defect or doping concentrations are some of the possible methods. Third, the scope of applications based on the photothermal behaviors of SNMs is required to expand. Up to now, the photothermal SNMs are mainly applied in the fields of solar vapor generation, biomedicine, and light‐responsive composites construction. For the further development of the applications of photothermal SNMs, some other fields should be concerned, such as photothermal catalysis, solar thermal storage, solar thermal power generation, etc. We hope this review can provide reference for the further development of SNMs in photothermal energy conversion.

## Conflict of Interest

The authors declare no conflict of interest.

## References

[1] B. D. Fahlman , Materials Chemistry 1, Springer, Mount Pleasant, MI, USA 2007, pp. 282–283.

[2] Y. Xia , Y. Xiong , B. Lim , S. E. Skrabalak , Angew. Chem., Int. Ed. Engl. 2009, 48, 60. 19053095 10.1002/anie.200802248 PMC2791829

[3] T. Trindade , P. O'Brien , N. Pickett , Chem. Mater. 2001, 13, 3843.

[4] X. Huang , Z. Yin , S. Wu , X. Qi , Q. He , Q. Zhang , Q. Yan , F. Boey , H. Zhang , Small 2011, 7, 1876. 21630440 10.1002/smll.201002009

[5] S. Y. Lim , W. Shen , Z. Gao , Chem. Soc. Rev. 2015, 44, 362. 25316556 10.1039/c4cs00269e

[6] B. Anasori , M. R. Lukatskaya , Y. Gogotsi , Nat. Rev. Mater. 2017, 2, 16098.

[7] X. Zou , Y. Zhang , Chem. Soc. Rev. 2015, 44, 5148. 25886650 10.1039/c4cs00448e

[8] H. I. Karunadasa , E. Montalvo , Y. Sun , M. Majda , J. R. Long , C. J. Chang , Science 2012, 335, 698. 22323816 10.1126/science.1215868

[9] L. Zhang , H. Wu , Y. Yan , X. Wang , D. Lou , Energy Environ. Sci. 2014, 7, 3302.

[10] A. S. Aricò , P. Bruce , B. Scrosati , J. M. Tarascon , W. van Schalkwijk , Nat. Mater. 2005, 4, 366. 15867920 10.1038/nmat1368

[11] P. Balaya , H. Li , L. Kienle , J. Maier , Adv. Funct. Mater. 2003, 13, 621.

[12] Q. Lu , W. Shi , H. Yang , X. Wang , Adv. Mater. 2020, 32, 2001544. 10.1002/adma.202001544 32935883

[13] W. Zhu , Y. Zhang , H. Zhang , H. Lv , Q. Li , R. Michalsky , A. A. Peterson , S. Sun , J. Am. Chem. Soc. 2014, 136, 16132. 25380393 10.1021/ja5095099

[14] M. Ferrari , Nat. Rev. Cancer 2005, 5, 161. 15738981 10.1038/nrc1566

[15] Z. Wang , Y. Ju , Z. Ali , H. Yin , F. Sheng , J. Lin , B. Wang , Y. Hou , Nat. Commun. 2019, 10, 4418. 31562357 10.1038/s41467-019-12142-4 PMC6765052

[16] Z. Wang , Z. Li , Z. Sun , S. Wang , Z. Ali , S. Zhu , S. Liu , Q. Ren , F. Sheng , B. Wang , Y. Hou , Sci. Adv. 2020, 6, 8733. 10.1126/sciadv.abc8733 PMC7695480 33246959

[17] T. He , C. Jiang , J. He , Y. Zhang , G. He , J. Wu , J. Lin , X. Zhou , P. Huang , Adv. Mater. 2021, 33, 2008540. 10.1002/adma.202008540 33645863

[18] M. Rycenga , C. M. Cobley , J. Zeng , W. Li , C. H. Moran , Q. Zhang , D. Qin , Y. Xia , Chem. Rev. 2011, 111, 3669. 21395318 10.1021/cr100275d PMC3110991

[19] B. Ni , Y. Shi , X. Wang , Adv. Mater. 2018, 30, 1802031. 10.1002/adma.201802031 30039573

[20] B. Ni , X. Wang , Chem. Sci. 2016, 7, 3978. 30155040 10.1039/c6sc00432f PMC6013797

[21] M. T. Pope , A. Miiller , Angew. Chem., Int. Ed. Engl. 1991, 30, 34.

[22] X. Wan , J. Wang , Z. Nan , Q. Wang , Sci. Adv. 2017, 3, 1701823. 10.1126/sciadv.1701823 PMC5630233 28989966

[23] S. Hu , H. Liu , P. Wang , X. Wang , J. Am. Chem. Soc. 2013, 135, 11115. 23837618 10.1021/ja403471d

[24] C. Teng , R. Zhai , Z. Li , X. Ma , L. Su , C. Chen , M. Cao , J. Yang , J. Wang , Carbon Trends 2021, 5, 100104.

[25] Q. Wang , D. O'Hare , Chem. Rev. 2012, 112, 4124. 22452296 10.1021/cr200434v

[26] J. Yu , B. R. Martin , A. Clearfield , Z. Luo , L. Sun , Nanoscale 2015, 7, 9448. 25963578 10.1039/c5nr01077b

[27] M. Chhowalla , H. S. Shin , G. Eda , L. J. Li , K. P. Loh , H. Zhang , Nat. Chem. 2013, 5, 263. 23511414 10.1038/nchem.1589

[28] J. Park , M. Kim , E. Cha , J. Kim , W. Choi , Sci. Rep. 2017, 7, 16121. 29170514 10.1038/s41598-017-16251-2 PMC5700996

[29] H. Liu , Y. Du , Y. Deng , P. D. Ye , Chem. Soc. Rev. 2015, 44, 2732. 25307017 10.1039/c4cs00257a

[30] J. Jiang , H. S. Park , J. Phys. D: Appl. Phys. 2014, 47, 385304.

[31] T. Lv , E. Zhang , Y. Yuan , Z. Fan , X. Ma , Z. Cheng , Y. Liu , Y. Gong , H. Zhao , Chem. J. Chin. Univ. 2019, 40, 2059.

[32] M. Zhao , Y. Huang , Y. Peng , Z. Huang , Q. Ma , H. Zhang , Chem. Soc. Rev. 2018, 47, 6267. 29971309 10.1039/c8cs00268a

[33] W. Pang , B. Shao , X. Tan , C. Tang , Z. Zhang , J. Huang , Nanoscale 2020, 12, 3623. 31989144 10.1039/c9nr09742b

[34] D. Rodriguez‐San‐Miguel , C. Montoro , F. Zamora , Chem. Soc. Rev. 2020, 49, 2291. 32182308 10.1039/c9cs00890j

[35] L. Xu , X. Zhou , W. Tian , T. Gao , Y. Zhang , S. Lei , Z. Liu , Angew. Chem., Int. Ed. 2014, 53, 9564. 10.1002/anie.201400273 25145927

[36] X. Huang , X. Qi , F. Boey , H. Zhang , Chem. Soc. Rev. 2012, 41, 666. 21796314 10.1039/c1cs15078b

[37] E. Fernandez , M. Boronat , J. Phys.: Condens. Matter 2019, 31, 013002. 30499451 10.1088/1361-648X/aaed84

[38] E. Schena , P. Saccomandi , Y. Fong , J. Funct. Biomater. 2017, 8, 19. 10.3390/jfb8020019 PMC5492000 28613248

[39] Q. Chen , S. He , F. Zhang , F. Cui , J. Liu , M. Wang , D. Wang , Z. Jin , C. Li , Sci. China Mater. 2021, 64, 510.

[40] X. Yang , L. Gao , Q. Guo , Y. Li , Y. Ma , J. Yang , C. Gong , C. Yi , Nano Res. 2020, 13, 2579.

[41] C. Tang , C. Song , Z. Wei , C. Liang , J. Ran , Y. Cai , X. Dong , W. Han , Sci. China Chem. 2020, 63, 946.

[42] C. Guo , X. Ma , B. Wang , Acta Chim. Sin. 2021, 79, 967.

[43] T. Wang , L. Zhao , K. Wang , Y. Bai , F. Feng , Acta Chim. Sin. 2021, 79, 600.

[44] S. Kumari , N. Sharma , S. V. Sahi , Pharmaceutics 2021, 13, 1174. 34452135 10.3390/pharmaceutics13081174 PMC8398544

[45] A. Bychkov , V. Simonova , V. Zarubin , E. Cherepetskaya , A. Karabutov , Appl. Sci. 2018, 8, 1931. 10.1364/AO.57.000C70 29714207

[46] Y. Wang , J. Zhang , W. Liang , H. Yang , T. Guan , B. Zhao , Y. Sun , L. Chi , L. Jiang , CCS Chem. 2021, 3, 2127.

[47] F. Tao , M. Green , A. V. Garcia , T. Xiao , A. T. Van Tran , Y. Zhang , Y. Yin , X. Chen , Appl. Mater. Today 2019, 17, 45.

[48] A. Ma , Y. Chen , Y. Liu , J. Gui , Y. Yu , Chem. Res. Chin. Univ. 2020, 36, 699.

[49] F. Xu , D. Weng , X. Li , Y. Li , J. Sun , CCS Chem. 2021, 3, 2494.

[50] C. Song , L. Hao , B. Zhang , Z. Dong , Q. Tang , J. Min , Q. Zhao , R. Niu , J. Gong , T. Tang , Sci. China Mater. 2020, 63, 779.

[51] W. Bian , Y. Huang , X. Xu , M. A. Ud Din , G. Xie , X. Wang , ACS Appl. Mater. Interfaces 2018, 10, 9407. 29468865 10.1021/acsami.7b18875

[52] J. He , H. Liu , B. Xu , X. Wang , Small 2015, 11, 1144. 25098755 10.1002/smll.201401273

[53] H. Liu , Z. Wu , J. Shao , D. Yao , H. Gao , Y. Liu , W. Yu , H. Zhang , B. Yang , ACS Nano 2017, 11, 2239. 28145697 10.1021/acsnano.6b08747

[54] M. V. Reddy , C. T. Cherian , K. Ramanathan , K. C. W. Jie , T. Y. W. Daryl , T. Y. Hao , S. Adams , K. P. Loh , B. V. R. Chowdari , Electrochim. Acta 2014, 118, 75.

[55] M. Chen , X. Shen , Q. Wu , W. Li , G. Diao , J. Mater. Sci. 2015, 50, 4083.

[56] W. F. Io , S. Yuan , S. Y. Pang , L. W. Wong , J. Zhao , J. Hao , Nano Res. 2020, 13, 1897.

[57] L. Zhou , Y. Tan , J. Wang , W. Xu , Y. Yuan , W. Cai , S. Zhu , J. Zhu , Nat. Photonics 2016, 10, 393.

[58] B. Ni , Q. Zhang , C. Ouyang , S. Zhang , B. Yu , J. Zhuang , L. Gu , X. Wang , CCS Chem. 2019, 1, 642.

[59] J. Liu , W. Shi , B. Ni , Y. Yang , S. Li , J. Zhuang , X. Wang , Nat. Chem. 2019, 11, 839. 31406324 10.1038/s41557-019-0303-0

[60] P. Wang , Y. Yang , J. Zhuang , X. Wang , J. Am. Chem. Soc. 2013, 135, 6834. 23611283 10.1021/ja403065z

[61] Y. Yang , Y. Yang , S. Chen , Q. Lu , L. Song , Y. Wei , X. Wang , Nat. Commun. 2017, 8, 1559. 29146895 10.1038/s41467-017-00850-8 PMC5691127

[62] S. Zhang , Y. Shi , T. He , B. Ni , C. Li , X. Wang , Chem. Mater. 2018, 30, 8727.

[63] Q. Lu , Y. Yang , J. Feng , X. Wang , Sol. RRL 2019, 3, 1800277.

[64] Q. Lu , B. Huang , Q. Zhang , S. Chen , L. Gu , L. Song , Y. Yang , X. Wang , J. Am. Chem. Soc. 2021, 143, 9858. 34156844 10.1021/jacs.1c03607

[65] J. Liu , S. Wang , N. Liu , D. Yang , H. Wang , H. Hu , J. Zhuang , X. Wang , Small 2021, 17, 2006260. 10.1002/smll.202006260 33373170

[66] S. Zhang , N. Liu , H. Wang , Q. Lu , W. Shi , X. Wang , Adv. Mater. 2021, 33, 2100576. 10.1002/adma.202100576 33904197

[67] S. Zhang , Q. Lu , B. Yu , X. Cheng , J. Zhuang , X. Wang , Adv. Funct. Mater. 2021, 31, 2100703.

[68] J. Liu , N. Liu , H. Wang , W. Shi , J. Zhuang , X. Wang , J. Am. Chem. Soc. 2020, 142, 17557. 32954726 10.1021/jacs.0c07375

[69] J. Liu , W. Shi , X. Wang , J. Am. Chem. Soc. 2019, 141, 18754. 31701740 10.1021/jacs.9b08818

[70] B. Akram , W. Shi , H. Zhang , S. Ullah , M. Khurram , X. Wang , Angew. Chem., Int. Ed. Engl. 2020, 59, 8497. 31573137 10.1002/anie.201910741

[71] B. Akram , B. Ni , X. Wang , Adv. Mater. 2020, 32, 1906794. 10.1002/adma.201906794 31773834

[72] H. Yang , D. Yang , X. Wang , Angew. Chem., Int. Ed. Engl. 2020, 59, 15527. 32378278 10.1002/anie.202004563

[73] J. T. Robinson , S. M. Tabakman , Y. Liang , H. Wang , H. S. Casalongue , D. Vinh , H. Dai , J. Am. Chem. Soc. 2011, 133, 6825. 21476500 10.1021/ja2010175

[74] F. Peng , F. Zhao , L. Shan , R. Li , S. Jiang , P. Zhang , Colloids Surf., B 2021, 198, 111467. 10.1016/j.colsurfb.2020.111467 33302151

[75] H. Lin , X. Wang , L. Yu , Y. Chen , J. Shi , Nano Lett. 2017, 17, 384. 28026960 10.1021/acs.nanolett.6b04339

[76] L. Cheng , J. Liu , X. Gu , H. Gong , X. Shi , T. Liu , C. Wang , X. Wang , G. Liu , H. Xing , W. Bu , B. Sun , Z. Liu , Adv. Mater. 2014, 26, 1886. 24375758 10.1002/adma.201304497

[77] T. Hu , L. Yan , Z. Wang , W. Shen , R. Liang , D. Yan , M. Wei , Chem. Sci. 2021, 12, 2594. 34164027 10.1039/d0sc06742c PMC8179329

[78] Z. Xie , Y. Duo , Z. Lin , T. Fan , C. Xing , L. Yu , R. Wang , M. Qiu , Y. Zhang , Y. Zhao , X. Yan , H. Zhang , Adv. Sci. 2020, 7, 1902236. 10.1002/advs.201902236 PMC7055570 32154070

[79] H. Bai , W. Yi , J. Li , G. Xi , Y. Li , H. Yang , J. Liu , J. Mater. Chem. A 2016, 4, 1566.

[80] X. Wu , Y. Li , G. Zhang , H. Chen , J. Li , K. Wang , Y. Pan , Y. Zhao , Y. Sun , Y. Xie , J. Am. Chem. Soc. 2019, 141, 5267. 30832477 10.1021/jacs.8b12928

[81] T. R. Gordon , M. Cargnello , T. Paik , F. Mangolini , R. T. Weber , P. Fornasiero , C. B. Murray , J. Am. Chem. Soc. 2012, 134, 6751. 22444667 10.1021/ja300823a

[82] J. Wang , Y. Li , L. Deng , N. Wei , Y. Weng , S. Dong , D. Qi , J. Qiu , X. Chen , T. Wu , Adv. Mater. 2017, 29, 1603730. 10.1002/adma.201603730 27862379

[83] P. Ying , M. Li , F. Yu , Y. Geng , L. Zhang , J. He , Y. Zheng , R. Chen , ACS Appl. Mater. Interfaces 2020, 12, 32880. 32589006 10.1021/acsami.0c09965

[84] P. Tao , G. Ni , C. Song , W. Shang , J. Wu , J. Zhu , G. Chen , T. Deng , Nat. Energy 2018, 3, 1031.

[85] G. Ni , G. Li , S. V. Boriskina , H. Li , W. Yang , T. Zhang , G. Chen , Nat. Energy 2016, 1, 16126.

[86] L. Zhou , Y. Tan , D. Ji , B. Zhu , P. Zhang , J. Xu , Q. Gan , Z. Yu , J. Zhu , Sci. Adv. 2016, 2, 1501227. 10.1126/sciadv.1501227 PMC4846456 27152335

[87] M. Ye , J. Jia , Z. Wu , C. Qian , R. Chen , P. G. O'Brien , W. Sun , Y. Dong , G. A. Ozin , Adv. Energy Mater. 2017, 7, 1601811.

[88] D. Ding , W. Huang , C. Song , M. Yan , C. Guo , S. Liu , Chem. Commun. 2017, 53, 6744. 10.1039/c7cc01427a 28589975

[89] H. Ghasemi , G. Ni , A. M. Marconnet , J. Loomis , S. Yerci , N. Miljkovic , G. Chen , Nat. Commun. 2014, 5, 4449. 25043613 10.1038/ncomms5449

[90] Y. Yang , R. Zhao , T. Zhang , K. Zhao , P. Xiao , Y. Ma , P. M. Ajayan , G. Shi , Y. Chen , ACS Nano 2018, 12, 829. 29301080 10.1021/acsnano.7b08196

[91] L. Zhang , B. Tang , J. Wu , R. Li , P. Wang , Adv. Mater. 2015, 27, 4889. 26184454 10.1002/adma.201502362

[92] Q. Chen , Z. Pei , Y. Xu , Z. Li , Y. Yang , Y. Wei , Y. Ji , Chem. Sci. 2018, 9, 623. 29629127 10.1039/c7sc02967e PMC5868306

[93] Y. Wang , N. Gong , Y. Li , Q. Lu , X. Wang , J. Li , J. Am. Chem. Soc. 2020, 142, 1735. 31880437 10.1021/jacs.9b11553

[94] H. Lin , S. Gao , C. Dai , Y. Chen , J. Shi , J. Am. Chem. Soc. 2017, 139, 16235. 29063760 10.1021/jacs.7b07818

[95] L. Wang , S. Guan , Y. Weng , S. M. Xu , H. Lu , X. Meng , S. Zhou , ACS Appl. Mater. Interfaces 2019, 11, 6267. 30672683 10.1021/acsami.8b20639

[96] X. Han , J. Huang , H. Lin , Z. Wang , P. Li , Y. Chen , Adv. Healthcare Mater. 2018, 7, 1701394. 10.1002/adhm.201701394 29405649

